# Exploring the influence of cultural orientations on assessment of communication behaviours during patient-practitioner interactions

**DOI:** 10.1186/s12909-017-0899-y

**Published:** 2017-03-21

**Authors:** Kyle J. Wilby, Marjan J. B. Govaerts, Zubin Austin, Diana H. J. M. Dolmans

**Affiliations:** 1 College of Pharmacy, Qatar University, PO Box 2713, Doha, Qatar; 2 School of Health Professions Education, Faculty of Health, Medicine and Life Sciences, Maastricht University, Universiteitssingel 60, 6229 ER Maastricht, Netherlands; 3 Leslie Dan Faculty of Pharmacy, University of Toronto, 144 College St, Toronto, ON M5S 3M2 Canada

**Keywords:** Culture, Patient-practitioner communication, Communication, Assessment, Medical education

## Abstract

**Background:**

Research has shown that patients’ and practitioners’ cultural orientations affect communication behaviors and interpretations in cross-cultural patient-practitioner interactions. Little is known about the effect of cultural orientations on assessment of communication behaviors in cross-cultural educational settings. The purpose of this study is to explore cultural orientation as a potential source of assessor idiosyncrasy or between-assessor variability in assessment of communication skills. More specifically, we explored if and how (expert) assessors’ valuing of communication behaviours aligned with their cultural orientations (power-distance, masculinity-femininity, uncertainty avoidance, and individualism-collectivism).

**Methods:**

Twenty-five pharmacist-assessors watched 3 videotaped scenarios (patient-pharmacist interactions) and ranked each on a 5-point global rating scale. Videotaped scenarios demonstrated combinations of well-portrayed and borderline examples of instrumental and affective communication behaviours. We used stimulated recall and verbal protocol analysis to investigate assessors’ interpretations and evaluations of communication behaviours. Uttered assessments of communication behaviours were coded as instrumental (task-oriented) or affective (socioemotional) and either positive or negative. Cultural orientations were measured using the Individual Cultural Values Scale. Correlations between cultural orientations and global scores, and frequencies of positive, negative, and total utterances of instrumental and affective behaviours were determined.

**Results:**

Correlations were found to be scenario specific. In videos with poor or good performance, no differences were found across cultural orientations. When borderline performance was demonstrated, high power-distance and masculinity were significantly associated with higher global ratings (*r* = .445, and .537 respectively, *p* < 0.05) as well as with fewer negative utterances regarding instrumental (task focused) behaviours (*r* = −.533 and − .529, respectively). Higher masculinity scores were furthermore associated with positive utterances of affective (socioemotional) behaviours (*r* = .441).

**Conclusions:**

Our findings thus confirm cultural orientation as a source of assessor idiosyncrasy and meaningful variations in interpretation of communication behaviours. Interestingly, expert assessors generally agreed on scenarios of good or poor performances but borderline performance was influenced by cultural orientation. Contrary to current practices of assessor and assessment instrument standardization, findings support the use of multiple assessors for patient-practitioner interactions and development of qualitative assessment tools to capture these varying, yet valid, interpretations of performance.

## Background

Patient-practitioner interactions are essential for the delivery and uptake of healthcare services worldwide [1]. Provision of diagnostic testing or treatments without effective and safe communication with patients leads to poor health outcomes and mistrust in health systems [1, 2]. Many definitions of culture exist, however it can be broadly defined as groups of people sharing similar values and beliefs systems [3, 4]. This may include similarities based on origin, gender, religion, sexuality, and socioeconomic status, among others. Cultural diversity, i.e. differing values and beliefs systems between individuals, is implicated as a contributing factor to communication and health disparities [5]. Given increasing globalization of health care (migration of health professionals and patients alike) and patient-practitioner communication taking place in multicultural settings, health professions education programs must teach and assess effective communication strategies that prepare students to work with patients from diverse ethno-cultural and linguistic backgrounds [6].

There are numerous challenges associated with addressing the effects of globalization on teaching and assessment. First, globalization of healthcare and health education now requires teachers and learners to work within multicultural environments and perhaps more importantly, assessment has to take place in multicultural settings [7]. More specifically, assessment settings may consist of assessors from different cultural backgrounds practicing in countries and cultures where they have neither learned nor practiced before. Secondly, cultural adaptation of assessment instruments and frameworks may be required to fit the local contexts and/or assessors may be required to use instruments and frameworks that do not match their personal conceptualizations of what constitutes effective communication [8]. These considerations can greatly affect assessment processes and pose risks to validity without proper understanding of how these cultural factors must be accounted for.

A widely used theoretical model in cross-cultural research is Hofstede’s cultural dimensions theory, which summarizes five domains, or ‘dimensions’, that attempt to account for a spectrum of values and beliefs relating to a particular culture [9]. The five cultural dimensions are power-distance, individualism-collectivism, masculinity-femininity, uncertainty avoidance, and Confucian dynamism or long-term orientation [10]. The definition and explanation of each dimension is summarized in Table 1.

**Table 1:** Cultural dimension definitions and characteristics [9]

Dimension	Explanation
Power-distance	Extent to which less powerful members of institutions and organizations within a country expect and accept that power is distributed unequally (refers to family, school, community, workplace, etc.)
Uncertainty avoidance	Extent to which the members of a culture feel threatened by uncertain or unknown situations and avoidance of such situations (expressed through nervousness and a need for predictability/rules)
Masculinity-femininity	Extent to which dominant values in society are masculine (in masculine societies gender roles are distinct whereas is feminine societies gender roles overlap)
Individualism-collectivism	Extent of ties between individuals (ties are loose in individualistic societies but integrated and cohesive in collectivist societies)
Long-term orientation	Extent of long or short-term orientation in life (long-term oriented people value persistence, thrift, sense of shame, and order; short-term oriented people value personal steadiness and stability, protecting oneself, respect for tradition, and reciprocation of greetings, favours, and gifts)

Findings from a recent study by Meeuwesen and colleagues (2009) suggest that Hofstede’s cultural dimensions explain communication preferences during patient-practitioner interactions across physicians and patients in Europe [11]. More specifically, patients’ and physicians’ cultural orientations influenced both instrumental (defined as orientation, psychosocial talk, asking questions, counselling) and affective (social talk, agreement, backchannelling) behaviours in patient-practitioner interactions. Instrumental behaviours can be seen as more task-focused, while affective behaviours relate more to socioemotional exchange [12]. Meeuwesen and colleagues found, for example, that practitioners in highly individualistic countries showed more affective behaviours and focused less on instrumental behaviours such as asking questions and counselling. Practitioners from more masculine vs. feminine countries equally showed more affective behaviours (social talk and agreements). Practitioners high on uncertainty avoidance, on the other hand, paid more attention to instrumental behaviours related to psychosocial talk, whereas those higher on power-distance gave more orientation to their patients (instrumental), gave more social talk (affective), yet less backchannelling (affective) [11]. Although these findings show a relationship between cultural orientations and communication behaviours in practitioners, any link between assessment of these behaviours and cultural orientations is unknown. Based on the communication preferences associated with cultural orientations, one might expect assessors to favour communication behaviours that match those specific to their own cultural orientation [13].

Drawing from Hofstede’s cultural dimensions theory, the purpose of this study is to explore cultural orientation as a potential source of assessor idiosyncrasy or between-assessor variability in assessment of communication skills. Hofstede’s theory has been criticized for equating culture with national identity [14]; however for the purposes of this study we are focusing on relating the cultural dimensions to individuals and not national populations as a whole. This strategy is supported by a previous study measuring cultural orientations at the individual level [15].

Research purposes are translated into two specific research questions:

1. How are cultural orientations associated with overall global assessment scores for 3 videos portraying different patient-practitioner interactions?
2. How are cultural orientations associated with assessors’ interpretation and evaluation of affective and instrumental communication behaviours?

## Methods

### Study design

This was a stimulated recall study evaluating patient-practitioner interactions, using videotaped interactions as the assessment platform. Stimulated recall was used to capture assessors’ cognitive processing during observation and evaluation of performance [16]. Verbal protocol analysis [17] was used to explore how assessors interpreted and valued affective and instrumental communication behaviours to arrive at judgments about performance.

### Context of the study

The study was conducted in Doha, Qatar at the College of Pharmacy at Qatar University. Qatar is a small affluent country bordering Saudi Arabia and the Arabic Gulf. The population is diverse, with local Qatari comprising only approximately 20% of the people residing in Qatar [18]. Expatriates comprise the majority of the population and come from all world regions, primarily South Asia, Philippines, Middle East, North Africa, and Western countries. Healthcare and education sectors display similar ethnic diversity of working professionals. As part of a national vision, these sectors are undergoing major reforms to be in line with North American and European models and standards [19]. The setting is representative of a region with high ethnic diversity.

### Participants

We enrolled practicing pharmacists and pharmacy educators with faculty or clinical appointments at the College of Pharmacy at Qatar University. Subjects were eligible for this study if they had experience assessing communication skills of pharmacists or pharmacy students during experiential training internships or campus-based objective structured clinical examinations (OSCEs). Convenience sampling was used by sending personalized emails to potential subjects that provided study objectives and procedures and sought interest in participation. If interest to participate was expressed, subjects were given more details regarding the study and were required to provide informed consent. Recruitment took place over 2 weeks until the target sample size of 25 subjects was obtained. This target was based on previous exploratory studies in qualitative research employing similar methods and analysis [20, 21].

### Pilot

A small pilot was completed prior to enrolling subjects for the larger study. The purpose of the pilot was to select videotaped patient-pharmacist interactions for assessment in the larger study, to refine study methods and interviewing techniques prior to study implementation. Three expert- assessors were purposively chosen as experienced communication evaluators, in order to provide accurate baseline assessments of communication behaviours as displayed in the videos and to determine performance levels of videotaped candidates. Three pilot videos were chosen for inclusion in the study. The only procedural change based on pilot data was to formalize stop points during the stimulated recall procedure every 30 s to ensure consistency between subjects.

### Measurement

Assessors’ cultural orientations were measured through use of CVSCALE (Individual Cultural Values Scale). CVSCALE is a validated questionnaire to measure cultural orientations on an individual level [15]. The scale consists of 26 statements that subjects rank on a 5-point Likert scale of agreement (power-distance, uncertainty avoidance, masculinity, individualism) or importance (long-term orientation). We excluded items for long-term orientation due to no known influence on affective or instrumental communication behaviours, as per Meeuwesen et al. [11].

Three videos served as the patient-practitioner interactions to be evaluated [22–24]. The duration of each video was between two and five minutes and each video documented an interaction between a patient and a pharmacist. Videos were carefully selected based on differing performance levels of the practitioner for both instrumental and affective communication behaviours as determined from performance level assessments and global rankings during the pilot. Video scenarios are summarized in Table 2. The study was capped at three videos, in order to ensure procedure time remained feasible for recruited participants (up to 1 h total).

**Table 2:** Descriptions of selected videos

	Video 1	Video 2	Video 3
Setting	Outpatient	Outpatient	Outpatient
Pharmacist description	Female pharmacist (30–40 years old)	Male pharmacist (30–40 years old)	Female pharmacist (20–30 years old)
Patient description	Elderly male patient	Angry mother of a 15 year old daughter	Female (20–30 year old) patient
Interaction type	Warfarin counselling	Response to mother’s concerns regarding daughter’s contraceptive	Counselling on a cholesterol-lowering medication
Details	The pharmacist gives a brief overview of warfarin, although she generally mentions most counselling points. She appears nervous and does not react to the patient’s verbal and nonverbal communication cues. However, she asks the patient good questions and provides opportunity for follow up.	The pharmacist responds to the angry mother by maintaining his composure and providing her with psychosocial advice regarding interacting with her daughter. He also acts within the laws and regulations of the Canadian context by not breaching patient confidentiality. The mother is visibly satisfied at the end of the interaction.	The pharmacist gives a poor performance with respect to gathering and providing information. She fails to clarify unclear points and consistently makes assumptions regarding the patient’s medical and medication history. She is friendly and personable (uses social talk) yet her tone is described as ‘unprofessional’ and occasionally ‘harsh’. She summarizes the interaction but globally fails to engage the patient during the interaction.
Pharmacists’ Instrumental Communication	Positive – Mixed (asks good questions and gives information but counselling lacks depth)	Positive (provides excellent information and rationale)	Negative (fails to ask questions and provide information to the patient)
Pharmacists’ Affective Communication	Negative (pharmacist deemed unconfident, demonstrated poor eye contact, and fails to engage patient)	Positive (demonstrated empathy, uses good nonverbal communication, shows understanding, but at times harsh in tone and word choice)	Mixed (friendly and personable yet occasionally gives harsh and unprofessional tone)

A 5-point Likert global assessment scale was used to capture assessors’ judgements regarding pharmacist performance in each video. The 5-point scale consists of 3 descriptors that focused on global performance. Assessors were allowed to give scores from 1 to 5 at 0.5 increments.

### Procedures

- Step 1:
  - Subjects completed the CVSCALE prior to meeting with the investigators.
- Step 2:
  - After a brief orientation to the process and assessment instruments, subjects were shown three videos separately in a random order. Subjects were told to focus assessments on ‘communication skills’ and ‘global performance’ and were allowed to take notes throughout. After each video finished, subjects were asked to rank the performance of the pharmacist according to the global assessment tool described above.
- Step 3:
  - After initial ranking of global performance, participants were asked to complete a 30–60 min stimulated recall interview. The same interviewer (KW) completed all interviews to ensure standardization. A second person was present in 13 of 25 interviews to monitor protocol methods and ensure validity of data obtained. Immediately following rating of performance, the voice recorder was turned on and subjects were asked to verbally explain their rationale for the ranking. Next, subjects re-watched the video while being voice recorded and were instructed to verbalize thoughts regarding the pharmacist’s performance at any point during the interaction. If nothing had been said for thirty seconds, the researcher stopped the video and asked if subjects had any comments from the previous segment. The same process was repeated for all three videos.

### Data analysis

Item scores for each dimension on CVSCALE were averaged to determine subject scores on each cultural dimension.

Immediately following interviews, transcripts of recordings were produced verbatim and were validated by a research assistant. Once all transcripts were produced, they were segmented into phrases by one investigator (KW) with each segment representing a single thought or idea. Then, each segment was assigned a coding category based on a pre-defined coding framework, developed from the study by Meeuwesen et al. [11] and confirmed using known instrumental and affective behaviours [12]. Table 3 presents the final coding framework and categories.

**Table 3:** Final coding framework per categories of instrumental and affective communication behaviours

Category	Codes
Instrumental	Identified problem Listens attentively Confirms problem and screens further Negotiates agenda Elicits history Uses open and closed questioning Facilitates responses Periodically summarizes Uses concise questions and comments Avoids jargon Establishes timeline Systematic/organized Summarizes to confirm understanding Transitions Logical sequencing Appropriate timing Shares thinking Explains rationale Chunks and checks Assesses starting point Asks pertinent questions Gives information Adheres to regulations Clarifies information Contracts with patient for next step Provides safety nets Checks understanding Summarizes session
Affective	Greets patient Introduces self Confirms identity Obtains consent Provides privacy Demonstrates respect and interest Positive demeanor Non-judgmental Empathetic Sensitive Professional Listens attentively without interrupting Reacts to verbal and non-verbal cues Encourages expression of feelings Eye contact Facial expressions Posture, movement Gestures Rate of speech Volume of speech Tone of speech Hesitation Confidence Assertiveness Maintains composure Friendly/Social talk

One investigator (KW) and one research assistant independently coded 10 transcripts. Coding was compared after each transcript and any discrepancies were resolved through discussion. At this point, it was found coding matched for >90% of each transcript and one investigator (KW) completed coding for the remaining 15 transcripts. For each participant, the numbers of statements per coded category (instrumental or affective behaviours) were calculated and transformed to percentages in order to correct for between-subject variance in verbosity and elaboration of answers.

### Statistical analysis

Descriptive statistics were used to summarize subject demographics, video scores, and category frequencies. To answer our first research question, we used the nonparametric Spearman’s Rank Correlation Coefficient to identify correlations between each cultural dimension score and global assessment scores per video. To answer our second research question, we used Spearman’s Rank Correlation Coefficient to determine correlations between cultural dimension scores and positive, negative, and total utterances of instrumental and affective behaviour per video. For all statistical analyses, we tested the null hypothesis of no correlation (rho = 0). Statistical significance was pre-defined at an alpha level of 0.05. All analyses were completed using SPSS Statistics v.22.

## Results

A total of 25 subjects (60% male) were recruited and completed the interview process for the full study post-pilot. All participants were pharmacists and had at least 3 years of practice experience. All (25/25) had experience assessing students in practice and 16/25 (64%) had experience assessing students in OSCE settings. All subjects were employed in Qatar (academic or practice settings) during the study period. Subjects came from fourteen countries of origin, which included Canada, Egypt, Fiji, Ghana, India, Jordan, Lebanon, Nigeria, Peru, Qatar, Somalia, Sudan, Syria, and the USA. Measured cultural orientations are given in Table 4. Overall assessors scored low on power-distance, high on uncertainty avoidance, and average on masculinity-femininity and individual-collectivism. The greatest variability between assessors was noted for masculinity-femininity.

**Table 4:** Cultural dimension scores as measured by CVSCALE

Dimension	Median (scale 1–5) (range)
Power-Distance (*n* = 25)	1.60 (1.0–3.2)
Uncertainty Avoidance (*n* = 25)	4.00 (2.80–4.80)
Masculinity-Femininity (*n* = 25)	2.25 (1.25–4.25)
Individualism-Collectivism (*n* = 25)	3.33 (2.33–4.33)

Table 5 presents correlations between assessors’ cultural orientations and global score ratings. Participants gave video 2 the highest global score (good instrumental and affective), followed by video 1 (good instrumental, poor affective), and lastly video 3 (poor instrumental, mixed affective). The cultural orientations of power-distance and masculinity-femininity were significantly associated with global assessments of video 3. Specifically, those scoring higher on power-distance and higher on masculinity gave higher scores for this video (*r* = .445 and *r* = .537, respectively). No other significant correlations were found between video scores and cultural orientations.

**Table 5:** Overall global assessment scores given by assessors and correlations with measured cultural dimensions

Video	Median (scale 1–5) (range)	Correlation with Power-Distance (r)	Correlation with Masculinity-Femininity (r)	Correlation with Uncertainty Avoidance (r)	Correlation with Individualism-Collectivism (r)
1 (*n* = 25)	3.0 (2.0–4.5)	−.091 (*p* = 0.664)	−.023 (*p* = 0.915)	.150 (*p* = 0.474)	.314 (*p* = 0.126)
2 (*n* = 25)	5.0 (3.5–5.0)	.064 (*p* = 0.761)	−.055 (*p* = 794)	.128 (*p* = 0.543)	−.092 (*p* = 0.660)
3 (*n* = 25)	2.5 (1.0–5.0)	.445* (*p* = 0.026)	.537* (*p* = 0.006)	.116 (*p* = 0.579)	.212 (*p* = 0.308)

*denotes statistical significance (*p* < 0.05)

r = Spearman’s Rank Correlation Coefficient

Table 6 presents correlations between assessors’ cultural orientations and utterance proportions of positive, negative, and total instrumental and affective communication behaviours. Table 6 shows that assessor interpretations and valuing of observed behaviours in videos 1 and 2 reflect practitioner performance on the videos and does not seem to be affected by assessors’ cultural orientations. Only one significant correlation was noted in video 1 (Table 6). Masculine assessors were associated with giving less positive utterances of affective behaviours (*r* = −.396). However, these utterances only accounted for 1.5% of total assessor utterances on this video, which precludes interpretation of this result. No associations were significant in video 2.

**Table 6:** Overall percentages of utterances per category and correlations between utterance proportions and cultural dimensions of assessors

Variable	Overall percentage of utterances per category (SD)	Correlation with Power-Distance (r)	Correlation with Masculinity-Femininity (r)	Correlation with Uncertainty Avoidance (r)	Correlation with Individualism-Collectivism (r)
Video 1 Instrumental					
Positive	9.56% (0.07)	−.236	−.025	.135	−.064
Negative	24.1% (0.18)	.167	−.015	−.006	−.115
Total Instrumental	33.7% (0.17)	−.100	−.066	.028	−.207
Video 1 Affective					
Positive	1.54% (0.04)	−.360	−.396*	.127	−.032
Negative	64.8% (0.17)	−.067	.120	−.067	.204
Total Affective	66.3% (0.18)	−.100	.066	−.028	.207
Video 2 Instrumental					
Positive	33.7% (0.18)	.063	.024	.056	−.022
Negative	5.74% (0.12)	.150	.078	−.226	−.017
Total Instrumental	39.4% (0.17)	.195	.199	−.031	.064
Video 2 Affective					
Positive	49.9% (0.17)	.030	−.027	−.267	−.126
Negative	10.2% (0.15)	−.066	−.307	.219	.189
Total Affective	60.2% (0.18)	−.194	−.191	.044	−.050
Video 3 Instrumental					
Positive utterances	7.62% (0.16)	.372	.363	.117	.070
Negative utterances	39.9% (0.20)	−.533*	−.529 *	.179	−.243
Total Instrumental	47.5% (0.19)	−.247	−.343	.325	−.081
Video 3 Affective					
Positive utterances	10.1% (0.13)	.354	.441*	−.062	.121
Negative utterances	42.4% (0.20)	.104	.117	−.205	.015
Total Affective	52.5% (0.19)	.247	.343	−.325	.081

*denotes statistical significance (*p* < 0.05)

r = Spearman’s Rank Correlation Coefficient

Table 6 also shows that assessor interpretations and valuing of observed behaviours in video 3 also generally reflected practitioner performance (Table 2). Table 6 furthermore shows that, for video 3, assessors high on power distance and masculinity gave less negative utterances of instrumental behaviours (*r* = −.533 and − .529, respectively). Conversely, assessors high on masculinity were associated with giving more positive utterances of affective behaviours displayed in this particular video (*r* = .441).

## Discussion

This stimulated recall study with verbal protocol analysis attempted to answer two research questions pertaining to the effect of an assessor’s cultural orientations on assessment of communication behaviours. We found that assessors scoring high on power-distance and masculinity provided fewer negative utterances regarding instrumental behaviours, resulting in higher overall scores on an interaction deemed to demonstrate borderline performance (poor instrumental and mixed affective behaviours). These findings support the notion that cultural orientations can influence assessment of communication behaviours and have several implications for assessment in settings of high cultural diversity.

We found the influence of culture on assessment is likely interaction and context or scenario dependent, and more prevalent within borderline performances. This was demonstrated through significant correlations for global scores and assessor cognitive processes on one of three video interactions (video 3) that demonstrated borderline performance, as compared to the other two interactions or videos (videos 1 and 2). Video 2 depicted a practitioner performing very well on both instrumental and affective communication behaviours, while video 1 depicted a practitioner with good instrumental but poor affective behaviours. Assessors were more likely to agree on these videos, probably due to the explicit nature of the performance levels. However, the performance levels on video 3 were less clear and therefore could be more open to discrepancies in assessor judgements. As such, cultural orientations may have had a greater role in influencing both scoring and associated judgments of these behaviours.

The results obtained from video 3 suggest that assessors higher on power-distance and masculinity may prefer closed communication behaviours during patient-practitioner interactions. As described in Table 1, this interaction portrayed a young female pharmacist interacting with a young adult female patient regarding a new cholesterol-lowering medication. The pharmacist barely provided any information regarding the medication and its associated benefits and risks. Furthermore, the pharmacist did not engage the patient, due to which the communication was not patient-centered, and primarily used closed-ended questions. As open communication and patient-centered care are purported as preferred practice in Western settings [25], it is interesting that countries known to be high on power-distance are largely non-Western in nature (Arab, Asian, and Central American states) [9]. It is possible, therefore, that open, patient-centered communication styles may be more difficult for assessors originating and trained in these countries to interpret according to Western standards.

One important finding of this study is that assessors appeared to value communication behaviours that match their own communication preferences, and these differed amongst assessors themselves within the multicultural setting of this study. In particular, the findings on video 3 were in line with the results of Meeuwesen et al. [11] in the sense that assessors high on power-distance potentially rewarded social talk, as exemplified on video 3 (Table 2). Similarly, more masculine assessors in our study rewarded affective (socio-emotional) behaviours on video 3 positively. Our findings with respect to assessor differences are in line with assessor cognition literature, suggesting that assessors hold differing perspectives and each bring varying yet valid interpretations of candidate performance [26]. Obviously, if assessment is to remain a responsibility of a trained professional observing the interaction, it can be argued that multiple assessors with varying cultural backgrounds may be a preferred strategy to capture judgments from differing perspectives. More importantly, however, our findings illustrate the need to include teachers and trainers from various cultural backgrounds in training of communication skills, to promote student learning, to ensure that students receive feedback from various perspectives to develop adaptive communication behaviours and are well-prepared for practice in multicultural settings. Overall, our findings confirm that the perspective of the communication receiver is crucial in assessment of communication skills. This immediately raises the question of who is best suited to assess communication behaviours. In the end, it is the patient who ultimately receives communication and can deem whether or not it was effective for them. Solely relying on assessor interpretations, rather than the communication receiver’s (patient) interpretation, is not appropriate if assessors are not able to understand how the communication is influencing the receiver’s experience. Findings from our study therefore emphasize the key role of the patient in assessment of practitioner’s competence.

The results of our study thus have several implications for assessment of communication within patient-practitioner interactions, as well as for future research. Findings of this study reflect variations in the way performance can be understood, experienced, and interpreted in multicultural settings. Being aware of different interpretations of communication behaviours can provide valuable feedback for learners and help to better determine pass-fail decisions in both low and high stakes assessment. As indicated above, this suggests there may be a need in these settings to use multiple assessors with differing backgrounds to gain greater perspective on the communication behaviours exhibited. It also suggests that patients could be engaged in communication assessment, as they are the ultimate receiver of communication and judgments may provide more in depth feedback than what observing assessors can provide.

One may argue that assessor-training programs could be implemented to better ‘standardize’ assessments and provide a frame of reference according to communication norms deemed best practices within the local context. Assessor training programs have been previously implemented in attempts to increase the reliability of assessments by decreasing inter-assessor variability [26]. The problem with this approach, however, is that it does not account for these differing perspectives that could in fact be valid interpretations of performance. Based on our findings we suggest, therefore, assessor training should address cultural considerations from the patient perspective, in order to increase assessor cultural awareness and improve cultural sensitivity in assessment practices. Assessors must develop this expertise in order to adequately assess student adaptability and effectiveness of student communication behaviours within and across various contexts.

Our findings furthermore demonstrate a need to review assessment instruments for communication skills in multicultural settings. Rather than using rating scales to quantify student performance, it may be more beneficial to use qualitative or mixed methods techniques such as (standardized) narratives to gain a deeper understanding of student abilities and how communication behaviours are interpreted during assessment. This approach could also promote provision of rich feedback when used in a formative manner. Additionally, if multiple assessors are used or patients are engaged in the assessment process, this technique will allow students to have greater understanding of their performance from multiple, differing perspectives. Credibility of this approach, especially for determination of pass-fail decisions, should be evaluated in future studies.

The results of this study support future research initiatives in the area of culture and assessment. Our results demonstrate potential cultural influences on assessment, especially for students of borderline performance. Introduction of high stakes performance-based assessments in settings of great cultural diversity may be prone to discrepancies in pass-fail decisions and future studies should explore assessment methods and tools that can ensure defensible and robust decision making in these complex assessment settings. The effectiveness of the strategies we have outlined, including the use of multiple assessors and qualitative assessment techniques, should be an immediate research priority. Finally, the association of cultural orientations with both scores and cognitive processes generates a hypothesis that provision of feedback following direct observation of communication behaviours likely differs based on cultural background of assessors. This finding justifies further study on the impact of culture on formative assessment, feedback and use of feedback in development of communication skills.

Our findings must be interpreted in light of the limitations of our study. First, our sample size of assessors was small (*n* = 25). This likely limited our power to detect significant correlations with cultural dimensions, which may have led to type II errors. The small sample size also resulted in minimal distribution across some of the cultural dimensions, which again may have resulted in a homogenous sample and limited the ability to detect meaningful correlations outside of power-distance and masculinity. Secondly, only three patient-practitioner interactions were evaluated. As no previous information was available to guide choice of interactions, we decided to choose based on performance levels with respect to instrumental and affective communication behaviours, identified by expert-assessors. Based on findings from our study, however, future studies should choose interactions of borderline performance, as these interactions are likely more prone to variations explained by differing cultural beliefs. Finally, this study evaluated the broad categories of instrumental and affective communication. There are many specific communication behaviours, though, that may be interpreted and assessed differently within various cultural contexts (eye contact, confidentiality, physical touch) and these warrant further investigation. However, in light of these limitations, this study is the first to explore the effect of cultural orientations on assessment of communication behaviours. It generates meaningful practical implications for assessment settings high in cultural diversity, as well as suggestions for further research on effects of increasing globalization on health care professional training and assessment.

## Conclusions

The results of our exploratory study give greater understanding to the effect of culture on assessment of communication skills. Assessors’ cultural dimensions of power-distance and masculinity may influence assessor ratings, interpretations, and evaluations regarding communication behaviours in patient-practitioner interactions of borderline performance levels. This finding may have major implications on definitions of ‘accurate’ or ‘correct’ communication behaviours, student pass-fail decisions, and learner feedback. Contrary to current assessment practices of standardization and assessor calibration, these results support the use of multiple assessors with varying cultural backgrounds in multicultural settings, as well as further investigation into the use of qualitative or mixed quantitative-qualitative assessment methods. The results also warrant investigation into whether or not the communication receiver (i.e. patient) should be involved in the assessment process. Future studies should address these considerations, in order to better understand the effects of culture on assessment of communication skills, the effectiveness of assessment practices within multicultural settings, and any impact of assessment on patient care and health outcomes.
